# Frontal haemodynamic responses in depression and the effect of electroconvulsive therapy

**DOI:** 10.1177/0269881119858313

**Published:** 2019-06-25

**Authors:** Darragh Downey, Sabrina Brigadoi, Liam Trevithick, Rebecca Elliott, Clare Elwell, R Hamish McAllister-Williams, Ian M Anderson

**Affiliations:** 1 Faculty of Biology, Medicine and Health, The University of Manchester, Manchester, UK; 2 Biomedical Optics Research Laboratory, University College London, London, UK; 3 Department of Developmental and Social Psychology, University of Padova, Padova, Italy; 4 School of Psychology, Newcastle University, Newcastle upon Tyne, UK; 5 Northumberland Tyne and Wear NHS Foundation Trust, Newcastle upon Tyne, UK; 6 Neuroscience and Psychiatry Unit, The University of Manchester, Manchester, UK; 7 Institute of Neuroscience, Newcastle University, Newcastle upon Tyne, UK

**Keywords:** Electroconvulsive therapy, depression, functional near-infrared spectroscopy, verbal fluency, working memory

## Abstract

**Background:**

Reduced frontal cortex metabolism and blood flow in depression may be associated with low mood and cognitive impairment. Further reduction has been reported during a course of electroconvulsive therapy but it is not known if this relates to mood and cognitive changes caused by electroconvulsive therapy.

**Aims:**

The purpose of this study was to investigate frontal function while undertaking cognitive tasks in depressed patients compared with healthy controls, and following electroconvulsive therapy in patients.

**Methods:**

We measured frontal haemodynamic responses to a category verbal fluency task and a working memory N-back task using portable functional near infra-red spectroscopy (fNIRS) in 51 healthy controls and 18 severely depressed patients, 12 of whom were retested after the fourth treatment of a course of electroconvulsive therapy. Mood was assessed using the Montgomery Åsberg Depression Rating Scale and cognitive function using category Verbal Fluency from the Controlled Oral Word Association Test and Digit Span backwards.

**Results:**

Compared to healthy controls, depressed patients had bilaterally lower frontal oxyhaemoglobin responses to the cognitive tasks, although this was only significant for the N-Back task where performance correlated inversely with depression severity in patients. After four electroconvulsive therapy treatments oxyhaemoglobin responses were further reduced during the Verbal Fluency task but the changes did not correlate with mood or cognitive changes.

**Discussion:**

Our results confirmed a now extensive literature showing impaired frontal fNIRS oxyhaemoglobin responses to cognitive tasks in depression, and showed for the first time that these are further reduced during a course of electroconvulsive therapy. Further research is needed to investigate the biology and clinical utility of frontal fNIRS in psychiatric patients.

## Introduction

Cortical function, as measured by cerebral metabolism and blood flow, has been reported to be impaired in major depressive disorder (MDD) (Fitzgerald et al., 2008; Nikolaus et al., 2000) and this may be related to both lowered mood and to cognitive impairment (Mayberg, 2003). Effective treatment with antidepressants has been associated with normalisation of pre-treatment abnormalities (Fitzgerald et al., 2006, 2008) suggesting that these are state-dependent. Functional near-infrared spectroscopy (fNIRS) is a portable imaging method that allows measurement of changes in tissue concentrations of oxyhaemoglobin (HbO) and deoxyhaemoglobin (HbR) and has been used as a putative brain imaging methodology for measuring cortical function with the potential to investigate underlying brain mechanisms of psychiatric disorders in clinical settings and to aid diagnosis (Ho et al., 2016). Increases in HbO and decreases in HbR can be detected in response to cognitive tasks (Irani et al., 2007) and these are believed to reflect neuronal activation through the mechanism of neurovascular coupling (Hillman, 2014). Using fNIRS, blunted HbO responses have consistently been found over frontal regions in MDD individuals compared with healthy controls during verbal fluency tasks, and, based on fewer studies, the same has been found with other tasks such those probing working memory (Zhang et al., 2015). Evidence for a relationship between fNIRS-measured frontal haemodynamic responses to a verbal fluency task and depression severity is conflicting (Akashi et al., 2015; Kawano et al., 2016; Kito et al., 2014; Liu et al., 2014; Nishida et al., 2017; Ohta et al., 2008; Satomura et al., 2019), but some studies have found a larger haemodynamic response is associated with greater clinical improvement to subsequent treatment (Masuda et al., 2017; Satomura et al., 2019; Tomioka et al., 2015) suggesting that the abnormality may be clinically relevant.

Electroconvulsive therapy (ECT) is a rapidly effective acute antidepressant treatment (UK ECT Review Group, 2003), but during the treatment course causes large temporary impairments in many aspects of cognition such as memory, executive function and verbal fluency; in contrast other aspects such as verbal working memory are not significantly affected (Semkovska and McLoughlin, 2010). The most consistent effect of ECT on cerebral metabolism is a further decrease in the frontal cortex during a treatment course (Nobler and Sackeim, 2008; Schmidt et al., 2008) which may be related to both the mood improvement and the cognitive impairment seen with ECT (Nobler et al., 1994, 2001), but the picture is far from clear.

The aims of this study were to investigate the effects of depression and of ECT on frontal haemodynamic responses measured using fNIRS, and their relationship to depression severity and the cognitive side effects of ECT.

We hypothesised (a) that depressed patients receiving ECT would show impaired frontal HbO responses to verbal fluency and working memory tasks compared with healthy controls, with the degree of impairment related to depression severity, and (b) during a course of ECT haemodynamic responses to the two tasks would be differentially affected – responses to the verbal fluency task would be further reduced in line with the impaired performance previously reported during a course of ECT, whereas responses to a working memory task would be unaffected, reflecting the sparing of this cognitive function by ECT.

## Methods

### Study design and participants

This was a sub-study of a multicentre, randomised, placebo-controlled trial of ketamine as an adjunct to ECT in severely ill depressed patients (Anderson et al., 2017b and see Procedures below) in seven Trusts in the North of England with fNIRS carried out in two centres (Manchester and Newcastle). Recruited patients had a diagnosis of a unipolar or bipolar moderate or severe major depressive episode by DSM-IV criteria and had consented to have ECT. Other inclusion criteria were age ⩾18 years, ability to give valid consent with a verbal intelligence quotient (IQ) equivalent to ⩾85 (Wechsler, 2001), sufficient fluency in English to validly complete neuropsychological testing, and deemed medically fit to receive ketamine by an anaesthetist. Main exclusion criteria were ECT in the last three months, detention under the Mental Health Act (1983, as amended in 2007), a primary psychotic or schizoaffective disorder, current primary obsessive compulsive disorder, anorexia nervosa, or history of drug or alcohol dependence (DSM-IV criteria), organic brain disease or significant medical illness affecting neuropsychological function, <24 on the Mini Mental State Examination (MMSE) (Folstein et al., 1975), contraindication to ketamine, risk of pregnancy or breastfeeding. Healthy controls were recruited by advertisement and from relatives, and prospectively sex and age group matched as far as possible with patients in the main study. They were required to be in good physical health with no history of personal, or first-degree family, psychiatric disorder, significant medical illness, psychotropic medication or other medication that could interfere with neuropsychological function or an MMSE<24.

The procedures in this study complied with the Helsinki Declaration of 1975, as revised in 2008. Ethical approval was granted by the North West-Liverpool East Research Ethics Committee (REC Ref No. 12/NW/0021) on 25 January 2012. Clinical trial authorisation for the main study was given by the Medicines and Healthcare products Regulatory Agency (23148/0004/001-0001). All participants gave separate written informed consent to participate in the main study and the fNIRS sub-study. The study is registered with the International Standard Randomised Clinical Trial Number registry (ISRCTN14689382) and with the EU Clinical Trial register (EudraCT number 2011-005476-41).

### Procedures and assessments

Following baseline assessment, depressed patients were randomised to intravenous ketamine (a glutamate receptor antagonist) 0.5 mg/kg or saline augmentation of their anaesthetic induction agents and received ECT twice weekly based on standard clinical ECT protocols (Royal College of Psychiatrists, 2005). Electrode placement was predominantly bifrontotemporal using constant-current brief pulse (0.5 ms pulse width) stimuli to induce seizure with a treatment dose of 1.5 times threshold determined by stimulus titration in the first session treatment. The stimulus parameters and patient medication remained the same until after the fourth treatment unless requiring change for clinical reasons.

For the full details of range and timing of efficacy and neuropsychological assessments see Anderson et al. (Anderson et al., 2017b). Assessment included the Mini International Neuropsychiatric Interview (Sheenan et al., 1998) to determine diagnosis, the Massachusetts General Hospital Scale (MGHS) (Fava, 2003) assessing treatment in current episode, the Wechsler Test of Adult Reading (WTAR) (Wechsler, 2001) as a measure of premorbid intellectual functioning (IQ) and the MMSE (Folstein et al., 1975) to screen for cognitive impairment. Depression severity was assessed by the Montgomery Åsberg Depression Rating Scale (MADRS) (Montgomery and Asberg, 1979). Neuropsychiatric assessment consisted of tests of verbal and visual memory, attention and executive function (Anderson et al., 2017b). Tests reported here as counterparts to the fNIRS tasks are category verbal fluency, involving executive function and sematic memory, from the Controlled Oral Word Association Test (COWAT) (Benton et al., 1994), in which participants are asked to name as many examples as possible of a category of objects (animals or fruit and vegetables) in one minute, and clinical Digit Span backwards (Wechsler, 1981) involving attention and working memory storage and manipulation. In patients, neuropsychological assessments were carried out at least 24 h after an ECT treatment, and scheduled to occur during the ECT course after four ECT treatments, after the last ECT, and one and four months after the last ECT. Only assessments carried out at baseline and after four ECT treatments are reported as limited recruitment to the fNIRS sub-study and high attrition meant few patients received fNIRS assessment after this point. Healthy controls were tested on a single occasion. The fNIRS was carried out after the neuropsychological assessments at the same testing session.

### Near-infrared spectroscopy

Two purpose-built 24 channel optical topography systems (NTS optical imaging system, Gowerlabs Ltd, UK) utilised six light sources and two light detectors for each hemisphere. These were arranged in a star-like configuration (Supplementary Material Figure S1) which provided overlap between source-detector pairs (channels) to cover an approximately circular area of about 8 cm diameter on each side over the junction between dorsolateral and ventrolateral prefrontal cortex (Brodmann areas 44, 45, 46 and posterior parts of 9 and 10) (Supplementary Material Figure S2). The bilateral arrays used Velcro (hook and loop) pads positioned according to the electroencephalogram (EEG) 10/20 positioning system to ensure that the detectors were in a standard position just below the F_3_/F_4_ EEG placement, with the lowest sources lying on the Fp_1_-T_3_ and Fp_2_-T_4_ lines. Each light source emitted light at two wavelengths (780 and 850 nm) allowing differentiation between HbO and HbR given their different absorption spectra at these wavelengths. The distances between light sources and detectors were designed to include signal from superficial layers of the cerebral cortex (Irani et al., 2007) and each array consisted of twelve 35 mm and eight 43 mm channels; four anterior-posterior 50 mm channels were excluded due to insufficient signal. Data were acquired for each channel at a rate of 10 Hz and manually synchronised with the onset of behavioural tasks.

#### Imaging tasks

Two tasks were administered in a session taking 40–50 min altogether. In the verbal fluency task participants were shown a category on a computer screen and asked to vocalise a word that matched that category (e.g. boys names, jobs, games and sports, vegetables etc.) but instructed to minimise movement by limiting jaw movement and speaking softly/whispering. The task was paced to minimise inter-individual performance differences with word prompts every 3 s in a block lasting 30 s (nine prompts per block). Following a 30 s rest block there were 10 category naming blocks each followed by a 30 s rest block giving a total task duration of 10.5 min. Participants were observed to ensure that they were vocalising but no behavioural measures were recorded.

The working memory task was a simplified blocked N-Back task involving attention, storage and updating of working memory (Shelton et al., 2009). Participants were asked to respond by pressing a key when a letter presented on a screen was the same as one presented the letter before last (i.e. two-back) following a brief practice session. A total of 17 letters were displayed in each 30 s block, with four repeated (target) letters per block, followed by a 30 s rest block. Following an initial 30 s rest block, there were 10 two-back blocks each separated by 10 rest blocks making the task last 10.5 min. The number of correct responses (as a percentage of the total number of targets) was recorded.

### Power calculation and analysis

The original aim was to recruit 100 patients and 50 matched healthy controls to the fNIRS substudy which would have given an 80% power to detect a moderate effect size of 0.5 between groups with a two-sided alpha level of 0.05. Difficulties in recruitment to the main study (79 rather than 160 patients), geographical limitations in transporting the imaging systems, and the severity of illness limiting willingness to volunteer to additional tasks meant that only 18 patients were recruited.

Baseline and behavioural data were analysed in SPSS 22 (www.IBM.com) using unpaired *t*-tests or chi squared tests as applicable.

fNIRS data were analysed with the Homer2 NIRS processing package (Huppert et al., 2009) based in Matlab (Mathworks.com). For each participant, channels that measured a very low optical intensity (below the noise level of the device, e.g. due to poor optode-skin contact) were discarded from the analysis (occurring in less than 5% of the 20 included channels). Intensity data were then converted into optical attenuation data. Motion artefacts were identified in each channel as time points exceeding selected changes in amplitude (AmpThresh) or standard deviation (StdThresh) over a period of 0.5 s. The choice of these parameters is data-dependent (see Cooper et al., 2012), and a compromise between the number of motion artefacts identified in noisy channels and those identified in less noisy channels. In this study, StdThresh=10, and AmpThresh=0.5 for the healthy control; StdThresh=6.5, and AmpThresh=0.5 for the patient baseline N-Back task; StdThresh=8, and AmpThresh=0.5 for the patient baseline Verbal Fluency (VF) task and mid-ECT data of the N-Back task; StdThresh=9, and AmpThresh=0.5 for the patient mid-ECT data of the VF task. Note that different thresholds were employed to accurately detect motion artifacts in each group, while the subsequent motion correction step was applied equally to all groups. The identified motion artefacts were corrected by applying a spline interpolation algorithm (Scholkmann et al., 2010) which models the artefact with a cubic spline interpolation, subtracts it from the original signal and then corrects for the baseline shift caused by the subtraction. Since some of the motion artefacts might still be present in the data even after this correction step, trials with remaining motion artefacts were rejected. A band-pass filter (third-order Butterworth filter) with cut-off frequencies of 0–0.5 Hz was applied to the data to reduce high frequency noise. The filtered optical attenuation data were finally converted into concentration changes by applying the modified Beer-Lambert law (Delpy et al., 1988) assuming a differential pathlength factor of 6.26 (Duncan et al., 1996).

**Figure 1.**
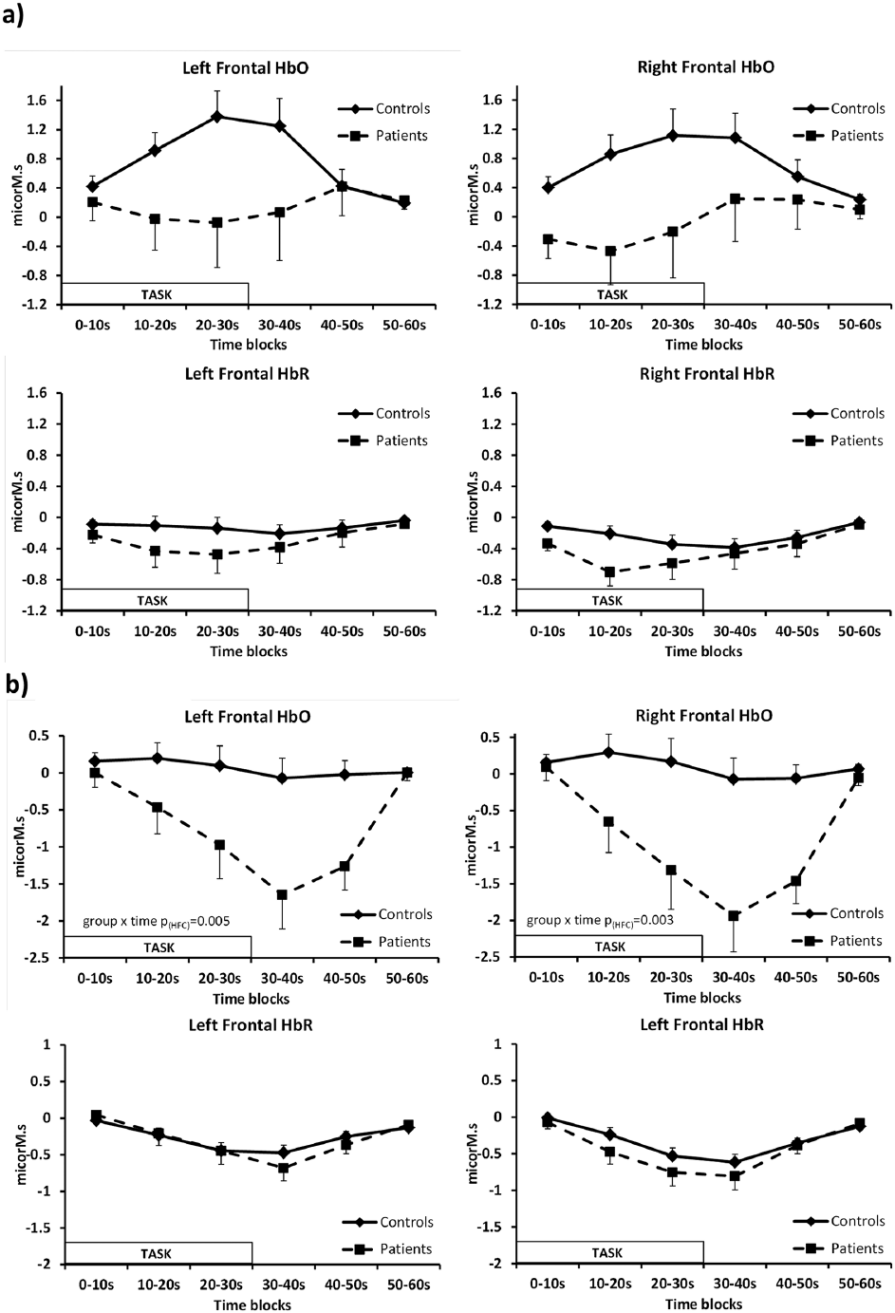
Right and left frontal oxyhaemoglobin (HbO) and deoxyhaemoglobin (HbR) responses for patients and controls over time. (a) Verbal Fluency task (HbO group×time *F*_(5,320)_=2.519, *p*(HFC)=0.08), (b) N-Back task (HbO group×time *F*_(5,325)_=6.002, *p*(HFC)=0.002). Values are mean±standard error of the mean (SEM).

Mean haemodynamic responses were calculated by block-averaging all trials in an interval from five seconds before stimulus presentation to 60 s after stimulus presentation (hence containing both the 30 s task period and the 30 s rest period). Mean haemodynamic responses were baseline corrected by subtracting the mean value in the –5 s to 0 s interval. This produced one mean haemodynamic response for each channel in each participant which was divided into six Blocks of 10 s (0–10 s, 10– 20 s, 20–30 s etc.); Blocks 1–3 were recorded during the task and Blocks 4–6 during the rest period (Supplementary Material Figure S3). The integral of the mean haemodynamic response in each of these Blocks was computed (Block area under the curve (AUC)) for analysis. Given that haemodynamic response to neuronal activation takes some time to reach its peak, the onset of the haemodynamic response during the active task occurs during Block 1, Blocks 2 and 3 encompass the period of greatest sustained response during the task, Block 4 the offset of the response and Blocks 5 and 6 the return of blood flow to rest level (as illustrated by the healthy control mean haemodynamic response in Figure 1). In preliminary analysis (Anderson et al., 2017a) the haemodynamic responses did not differ significantly in different areas of the array so the data were collapsed to cover two regions, one for each hemisphere, by calculating the mean of the channel values for each side (i.e. 6×35 mm and 4×43 mm channels per side). We report HbO and HbR measurements, the former regarded as the most sensitive measure and most commonly reported in tasks of cortical activation (Lu et al., 2015).

Analysis was carried out in SPSS 22 (www.IBM.com). The groups were compared (using patient baseline data) by analysing the six Block AUC values using a univariate repeated measures analysis of covariance (ANCOVA) with one between group factor (group) and two within-group factors (side and time). Age and sex may influence fNIRS signal (Herrmann et al., 2006; Yang et al., 2009) and were used as covariates given the nominal group differences although non-significant (see Results). To identify whether a haemodynamic response occurred in each group, a repeated measures analysis of variance (ANOVA) over time was carried out in healthy controls and patients at baseline separately. The effect of ECT was analysed in patients alone using a repeated measures ANOVA with three within-group factors (ECT (baseline vs mid-ECT), side and time). Patients received saline or ketamine during ECT treatments but analysis with drug as an additional factor is not reported here given no effect of ketamine on efficacy or cognition in the main study (Anderson et al., 2017b) and, as in the preliminary fNIRS analysis (Anderson et al., 2017a), it did not alter the results reported here. The Huyhn-Feldt correction (reported as *p*_(HFC)_) was applied to correct for sphericity violation and post-hoc exploration of individual hemispheres were carried out by repeated measures ANOVA or ANCOVA by side. For the verbal fluency task, the left hemisphere was identified *a priori* as a region of interest as the fNIRS haemodynamic response on the left has been shown to relate to functional magnetic resonance imaging (MRI) blood oxygen-level dependent (BOLD) signal change during this task (Murata et al., 2010). In order to examine the relationship between changes in HbO during the task and performance measures we used performance on COWAT category fluency for the verbal fluency task as no behavioural measures were recorded for this task, and the Digit Span backwards in addition to task performance for the working memory, N-Back task. Correlational analysis using Pearson’s correlation (*r*) was carried out between the overall HbO response (‘total AUC’ for each side calculated as the sum of the six Block AUC values) and planned behavioural comparisons: (a) left frontal haemodynamic responses to the verbal fluency task and depression severity (MADRS score), verbal fluency performance (COWAT category), and changes in these between baseline and mid-ECT, (b) left and right frontal haemodynamic responses to the N-Back task and MADRS score and behavioural performance on the N-Back task, Digit Span backwards, and changes in these between baseline and mid-ECT, (c) changes in HbO total AUC values both tasks from baseline to mid-ECT and changes in mood and behavioural scores for the respective tasks. Results are presented as mean (standard deviation (SD)) or ± SEM (with unadjusted degrees of freedom for clarity) and statistical significance if *p*<0.05.

## Results

The characteristics of patients and controls participating in the fNIRS study are shown in Table 1. Patients (16 unipolar, two bipolar depression) were severely depressed, all with melancholia, and on average had failed over four antidepressant drug treatments. All were medicated with two-thirds taking a combination of antidepressant and antipsychotic medication. Although patients were slightly younger, with a lower proportion of females, than healthy controls these differences were not significant. There was no significant difference in IQ, but patients had slightly, but significantly, lower MMSE scores, and much higher MADRS scores than healthy controls.

**Table 1.** Characteristic of patients and controls.

	Healthy controls (*n*=51)		Patients (*n*=18)	
	Mean (SD)	*n* (%)	Mean (SD) Median (IQR)	*n* (%)
Age (years)	56.2 (12.1)		51.6 (15.6)	
Sex (female ***n***, (%))		30 (59%)		8 (44%)
Ethnicity (white ***n***, (%))		50 (98%)		17 (94%)
Handedness (left/mixed)		2/4 (4%/8%)		1/0 (6%/0%)
Years in FT education	14.7 (3.3)		13.5 (3.2)	
Illness				
Bipolar disorder (***n***, %)		–		2 (11%)
Episode duration (months)	–		Median 20.5 (IQR 7.75–46.5)	
MGHS score	–		4.4 (3.2)	
IQ	110.7 (10.2)		108.6 (11.5)	
MMSE	29.8 (0.5)		29.0 (1.2)^a^	
MADRS	0.9 (1.5)		33.7 (7.3)^b^	
COWAT category fluency	23.2 (4.7)		15.7 (6.0)^b^	
Digit Span backward	4.9 ( 1.2)		3.8 ( 1.1)^b^	

COWAT: Controlled Oral Word Association Test; FT: full time; IQ: intelligence quotient; IQR: interquartile range; MADRS: Montgomery Åsberg Depression Rating Scale; MGHS: Massachusetts General Hospital Scale; MMSE: Mini Mental State Examination; SD: standard deviation.

a *p*<0.05, ^b^*p*⩽0.001 vs controls.

### Group comparison: Verbal Fluency task

#### Behavioural data

No behavioural data were collected during the fNIRS task but patients were markedly impaired on the COWAT category fluency (see Table 1). Given that the COWAT task reflects both the speed and number of words that are able to be retrieved its results may not reflect the patients’ performance during the fNIRS task which was paced in an attempt to reduce possible differences in performance between groups.

#### Haemodynamic responses

One patient did not undertake the Verbal Fluency task resulting in data being available from 17 patients and 51 healthy controls. The left frontal haemodynamic response was a little higher than on the right in controls but there were no significant time×side interactions (illustrated in Figure 1(a)). Group comparison showed non-significant trends for main effect of group (*F*_(1,64)_=3.562, *p*=0.06) and for interaction between time and group (*F*_(5,320)_=2.519, *p*_(HFC)_=0.08). Pre-planned analysis of the hemispheres individually showed a trend time×group interaction for the left hemisphere (*F*_(5,320)_=2.873, *p*_(HFC)_=0.06) but not for the right (*p*_(HFC)_>0.1). When the groups were analysed separately, a significant main effect of time was found for HbO in controls (*F*_(5,250)_=7.707, *p*_(HFC)_=0.001) with an increase during the Verbal Fluency task and a reduction towards baseline during the rest period (see Figure 1); no significant change over time was seen in patients (*p*_(HFC)_=0.7).

No main effects or interactions were found for HbR concentrations in the group comparison. There was no significant effect of time in each group considered separately.

#### Correlations

**Figure 2.**
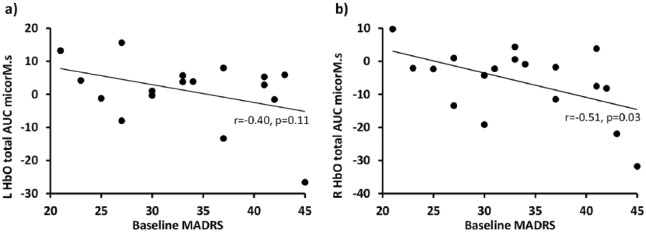
Correlations between depression severity (Montgomery Åsberg Depression Rating Scale (MADRS)) and haemodynamic responses (oxyhaemoglobin (HbO) total area under the curve (AUC)) in patients to (a) Verbal Fluency task in left hemisphere and (b) N-Back task in right hemisphere.

No significant correlations were found between haemodynamic responses and depression severity or COWAT performance at baseline or in their change after four ECT treatments, although at baseline left HbO total AUC had a negative correlation with MADRS score (*r*=−0.40, *p*=0.11, Figure 2(a)).

### Group comparison: N-Back task

#### Behavioural data

Behavioural data from the N-Back task were only available for 20 healthy controls and 13 patients due to technical problems at one site. All participants had been assessed on Digit Span backwards which has been shown to correlate significantly with the N-Back task (Shelton et al., 2009). In our total population it correlated modestly with N-Back accuracy in those who undertook both tasks: *r*=0.39, *p*=0.024 (controls *r*=0.26, *p*=0.28, patients *r*=0.31, *p*=0.3). When two outliers (one control, one patient) were excluded the correlation in the total population was greater overall *r*=0.57, *p*=0.001, weaker in controls (*r*=0.19, *p*=0.43) but stronger in patients *r*=0.78, *p*=0.003). Patients performed significantly less well on both tasks than healthy controls; the Digit Span backwards results are shown in Table 1 and fNIRS N-Back task accuracy was 87% (SD 19%) in healthy controls and 69% (SD 21%) in patients (*p*=0.02).

#### Haemodynamic responses

Data were available from 51 healthy controls and 18 patients for the N-Back task. The haemodynamic responses were similar on both sides for both groups (Figure 1(b)). In the comparison of the two groups over time there was a significant main effect of group (*F*_(1,65)_=8.951, *p*=0.004) and time (*F*_(5,325)_=8.456, *p*_(HFC)_<0.001) and an interaction between time and group (*F*_(5,325)_=6.002, *p*_(HFC)_=0.002) but there were no significant group×side interactions (*p*>0.4). When groups were analysed separately, healthy controls did not show a significant HbO response over time to the N-Back task (*p*_(HFC)_=0.3) but patients had a significant decrease in HbO signal during the task (time: *F*_(5,85)_=5.310, *p*_(HFC)_=0.008).

For HbR (Figure 1(b)) there was a main effect of time (*F*_(5,325)_=3.763, *p*_(HFC)_<0.016) but no significant main effect of group or interaction between time and group (*p*>0.5). Both groups showed a significant decrease in HbR during the task (effect of time: healthy controls *F*_(5,250)_=15.345, *p*_(HFC)_<0.001; patients *F*_(5,85)_=8.656, *p*_(HFC)_=0.001).

#### Correlations

In all participants taken together total HbO AUC correlated negatively with MADRS score (right hemisphere *r*=−0.47, *p*<0.001; left hemisphere *r*=−0.42, *p*=0.001) and positively with Digit span backwards (right hemisphere *r*=0.28, *p*=0.02; left hemisphere *r*=0.34, *p*=0.005) probably reflecting the group differences in these measures. In controls left hemisphere total HbO AUC correlated positively with Digit span backwards (*r*=0.28, *p*<0.05) and for patients right hemisphere total HbO AUC correlated negatively with MADRS score (*r*=−0.51, *p*=0.03, Figure 2(b)). In patients baseline HbO values did not correlate with severity or performance changes after four ECT treatments.

### Effect of ECT

#### Behavioural data

Data were available from 12 patients who received fNIRS tasks after four ECT treatments. A modest but significant reduction in the severity of depression after four ECT was seen with MADRS scores decreasing from 33.4 (SD 7.7) to 25.3 (SD 11.1) (*p*=0.02), with four patients (33%) going on to respond (⩾50% decrease in MADRS) at the end of the course of ECT and three (25%) remitting (final MADRS ⩽10). COWAT category fluency improved non-significantly from 14.1 (SD 5.7) to 15.3 (SD 5.0) (*p*=0.39) with no change in Digit Span backwards which was 3.8 (SD 1.3) both before and after four ECT (*p*=0.75).

#### Verbal Fluency task haemodynamic responses

**Figure 3.**
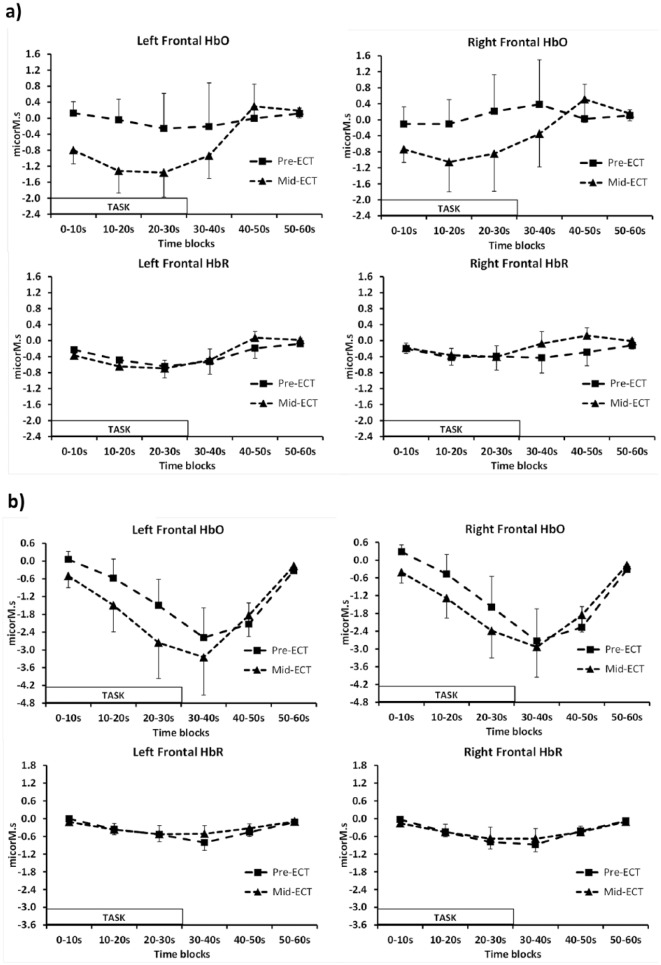
Right and left frontal oxyhaemoglobin (HbO) and deoxyhaemoglobin (HbR) responses for patients before (pre-electroconvulsive therapy (ECT)) and during ECT (mid-ECT). (a) Verbal Fluency task (HbO time×ECT *F*_(5,50)_=3.140, *p*(HFC)=0.045), (b) N-Back task (HbO time×ECT *F*_(5,50)_=2.315, *p*(HFC)=0.12). Values are mean±standard error of the mean (SEM).

Data were available for 11 patients before and during the ECT course. For HbO there was a significant interaction between time and ECT (*F*_(5,50)_=3.140, *p*_(HFC)_=0.045) with no significant main effects of ECT, time or ECT by side interactions (Figure 3(a)). Following ECT there was decrease in HbO signal during the task compared to a lack of change at baseline. Analysis of each side separately showed a trend time×ECT interaction for both hemispheres (*p*_(HFC)_<0.09 on the left and *p*_(HFC)_<0.06 on the right).

No main effects or interactions were found for HbR concentrations on the two occasions.

#### N-Back task haemodynamic responses

Data were available for 12 patients before and during the ECT course. Both HbO and HbR decreased during the task and returned to baseline in the rest periods, with a significant effect of time found (*F*_(5,55)_=6.134, *p*_(HFC)_=0.014 and *F*_(5,55)_=4.370, *p*_(HFC)_=0.025 respectively). The slightly lower HbO signal mid-ECT compared with baseline in response to the task was not significant (time×ECT interaction *F*_(5,55)_=2.315, *p*_(HFC)_=0.12) with no other significant main effects nor interactions (Figure 3(b)).

#### Correlations

No significant correlations were seen between HbO total AUC changes between baseline and mid-ECT for either task and changes in COWAT category verbal fluency, Digit Span backwards, N-Back accuracy or MADRS score.

## Discussion

We found that frontal HbO haemodynamic response to a working memory N-Back task measured using fNIRS was reduced in severely depressed patients compared to healthy controls, and that the response in patients was inversely related to depression severity. The results for haemodynamic responses to the Verbal Fluency task were similar but only at trend level. Performance on behavioural counterparts of the tasks was markedly impaired in patients compared with controls but did not consistently correlate with the haemodynamic response, apart from a modest positive relationship in controls between the right frontal HbO response and performance on Digit Span backwards. During a course of ECT in patients, the overall HbO response to the Verbal Fluency task was lower than before ECT, with the haemodynamic response to the N-Back task non-significantly lower. Category verbal fluency and working memory performance were not significantly affected by ECT and appeared unrelated to the haemodynamic responses.

There is a substantial body of evidence for a reduction in frontal fNIRS HbO response to verbal fluency tasks in depression, with a systematic review meta-analysis by Zhang et al. (Zhang et al., 2015) identifying 11 studies of which all reported impaired responses and a quantitative analysis of a sub-group showing a large standardised effect size (0.76). Further studies have overwhelmingly supported this result (e.g. Akashi et al., 2015; Akiyama et al., 2018; Fu et al., 2018; Hirano et al., 2017; Ma et al., 2017; Ohi et al., 2017; Tsujii et al., 2017) with a similar finding in bipolar depression (Fu et al., 2018; Kameyama et al., 2006; Ohi et al., 2017). The literature is therefore strikingly consistent with no reported studies showing normal responses. Our result is similar, although only at trend level, with only controls showing significant activation and the difference between groups showing a moderate mean standardised effect size difference of around 0.5 (estimated from the left-sided peak response illustrated in Figure 1(a)).

Zhang et al. (Zhang et al., 2015) also reported reduced fNIRS frontal responses to working memory tasks in MDD of a similar size to those found for verbal fluency and this has been replicated in a further study (Zhu et al., 2018). For this task we found a highly significant difference between groups with patients showing decreases in HbO during the task with a large peak difference mean effect size of about 0.8 (illustrated in Figure 1(b)). However, there was a different pattern of HbO signal change for the N-Back compared with the Verbal Fluency tasks in our study, with little change found in controls although a striking decrease found in patients. The reason for this is unclear but a similar reduction in frontal haemodynamic signal to a working memory task has been described in participants with mild cognitive impairment (Niu et al., 2013) with the authors suggesting that there may be an alteration in the regional pattern of activation in the working memory cortical network in these patients. Given that depression is consistently associated with cognitive impairment (Clark et al., 2009), as we have also reported in patients participating in the current study (Anderson et al., 2017a), it is possible that a similar mechanism is present here.

The majority of studies have found no link between depression severity and frontal haemodynamic response during verbal fluency tasks (Akashi et al., 2015; Kameyama et al., 2006; Kito et al., 2014; Ohta et al., 2008; Ohtani et al., 2015; Pu et al., 2008; Pu et al., 2012a; Satomura et al., 2019; Tomioka et al., 2015; Tsujii et al., 2014, 2016) but a minority have found a negative (Kawano et al., 2016; Nishida et al., 2017; Noda et al., 2012) or a positive (Liu et al., 2014) relationship. In addition, whether or not impaired frontal responses are state-dependent is unclear. Two studies of remitted depressed patients reported impaired frontal haemodynamic responses compared with controls using a verbal fluency task (Ikeda et al., 2013; Matsuo et al., 2005) while another found less impairment compared to controls in left frontal response in partially remitted patients compared with those currently depressed (Akiyama et al., 2018). A further study found no increase in response in spite of symptomatically effective treatment with antidepressants (Tomioka et al., 2015), however an increase back to healthy control levels was seen in another following successful treatment with ECT and the change correlated with clinical improvement (Hirano et al., 2017). Taken as whole, the weight of evidence appears against a direct relationship with depression severity in patients but the data are conflicting as to whether the abnormality persists after remission. Consistent with findings using the verbal fluency task, two studies of depressed patients using a working memory task also reported no relationship between HbO responses and depression severity (Pu et al., 2011, 2012b).

Our finding of greater haemodynamic responses in verbal fluency and working memory associated with lower severity of depression (Figure 2) – although only significant for the N-Back task – therefore appears at odds with the majority of the literature. It is possible that differences in patient characteristics between studies may be important. Many studies involve mildly depressed patients whereas our patients were nearly all severely ill and melancholic, and it has been previously reported that melancholic patients have smaller haemodynamic responses than non-melancholic patients (Tsujii et al., 2014). The finding that larger responses to a verbal fluency task is related to better response to subsequent treatment with antidepressants in three studies (Masuda et al., 2017; Satomura et al., 2019; Tomioka et al., 2015) also points to the fNIRS haemodynamic response potentially reflecting a biologically relevant aspect of depression.

We found that ECT further reduced the frontal HbO signal during the Verbal Fluency task as we hypothesised, with no significant effect during the N-Back task, although it was numerically lower in the latter and therefore we cannot confidently exclude an effect. This contrasts with antidepressant therapy where no change in HbO to a verbal fluency task was noted after 12 weeks of treatment (Tomioka et al., 2015), and with an ECT study which found normalised responses two weeks after completion of the ECT course (Hirano et al., 2017). The latter is not necessarily inconsistent with our findings as we carried out testing a mean of two days after a previous ECT treatment. It is well recognised that the adverse effects of ECT on cognitive tests rapidly improve when ECT is stopped with restoration or improvement over baseline function by two weeks following ECT (Semkovska and McLoughlin, 2010). Given that functional effects of ECT are temporary, the same may be true of any suppression of haemodynamic responses. While our results are consistent with a reduction in cerebral metabolism reported during an ECT course (Nobler and Sackeim, 2008; Schmidt et al., 2008) we were not able to show any correlation between HbO response changes during ECT and those in cognitive performance or mood. This may not be surprising given that other studies have failed to find any relationship between performance and haemodynamic responses with verbal fluency tasks (Kameyama et al., 2006; Kito et al., 2014; Nishida et al., 2017; Noda et al., 2012; Ohtani et al., 2015; Pu et al., 2008; Satomura et al., 2019; Tomioka et al., 2015; Tsujii et al., 2014; Wang et al., 2017) and also Hirano et al. (Hirano et al., 2017) did not find that verbal fluency performance changes correlated with haemodynamic changes following ECT. At present therefore the functional significance of the changes in HbO response that we found during a course of ECT is unclear, especially as we did not find that COWAT category verbal fluency or Digit Span backwards were impaired after four ECT treatments in our patients which was unexpected.

We included a working memory N-Back task as a proposed contrast to the Verbal Fluency task. Working memory activates a cortical network used in maintaining task-relevant information during a delay, involving rehearsal and goal-directed attention. This includes the bilateral dorsolateral and ventrolateral prefrontal cortex (D’Esposito, 2007; Owen et al., 2005) but does not share subcortical aspects of the network activated by verbal fluency tasks, such as the hippocampus (Whitney et al., 2009), which is hypothesised to be involved in cognitive impairment due to ECT (van Oostrom et al., 2018). We had proposed that this network might be relatively spared by ECT given the lack of impairment found in performance on working memory tasks following ECT, possibly due to its lack of a hippocampal component. Consistent with our hypothesis, ECT did not significantly decrease HbO responses to the N-Back task although numerically it was lower. We found a different pattern of haemodynamic changes to the Verbal Fluency and N-Back tasks, the reasons for which are not clear. However, the effects of both depression and ECT were in the same direction for both tasks, and not related to performance measures in patients. Given this, it is difficult to draw strong conclusions from a comparison of the results from the two tasks.

fNIRS has predominantly been used to investigate patients with depression but it is important to note that blunted frontal haemodynamic responses to verbal fluency tasks do not appear to be diagnosis-specific and have been reported in schizophrenia (Kinou et al., 2013; Ohi et al., 2017), bipolar disorder (Fu et al., 2018; Kameyama et al., 2006; Ohi et al., 2017) and anxiety disorders (Kawano et al., 2016; Ohta et al., 2008). Some studies (Kinou et al., 2013; Pu et al., 2008), but not all (Tsujii et al., 2014), have found a link between haemodynamic responses and social or global function. There appears to be specificity for type of task as haemodynamic responses to motor tasks do not differ from healthy controls (Kameyama et al., 2006; Suto et al., 2004), with mixed findings of normal or blunted responses to hyperventilation/carbon dioxide inhalation (Matsuo et al., 2002, 2005; Nishida et al., 2017). This suggests that fNIRS used with cognitive tasks may provide a biological measure relevant to psychiatric disorders, especially given the evidence that it may predict treatment response to antidepressants in depression (Masuda et al., 2017; Satomura et al., 2019; Tomioka et al., 2015).

A strength of our study is the well-characterised patient population and, arguably, the use of averaged data from multiple channels covering two circumscribed frontal regions, one over each hemisphere, decreases false positives associated with multiple-testing of individual channels. However, there are important methodological limitations. Problems in recruitment meant that our patient group was small, and although we were able to show effects of diagnosis and ECT we lacked power to be able to adequately explore links between haemodynamic response and clinical or behavioural measures. In addition reliance on performance measures derived from behavioural testing, which may not have reflected performance during the fNIRS tasks, reduces confidence in our correlational analysis with haemodynamic responses, therefore making it difficult to know to what degree task performance might have influenced the results. The patients were medicated and we cannot exclude an effect of drugs on haemodynamic responses although this appears unlikely to be an important factor as impaired haemodynamic responses have been reported in drug-free depressed patients (Masuda et al., 2017; Tomioka et al., 2015) with no change after prospective antidepressant treatment (Tomioka et al., 2015), and our patients remained on the same medication when tested following ECT. An important limitation of the fNIRS technique used in our study is that the relative contribution of scalp and brain sources of the haemodynamic signal measured is unknown. Arrays with longer path-length source-detector pairs measure changes in haemochrome concentrations in tissues that include extra-cerebral tissues and superficial cerebral cortex (Brigadoi and Cooper, 2015). Studies using fNIRS consistently detect a broader frontal activation to verbal fluency tasks than the predominantly left-sided brain areas detected using fMRI (Allen and Fong 2008; Pihlajamaki et al., 2000; Whitney et al., 2009) and Takahashi et al. (Takahashi et al., 2011) reported that longer path-length HbO signal changes to a verbal fluency task correlated with changes in scalp blood flow and shorter path-length channels measuring extra-cerebral signal. However another study found that left frontal fNIRS responses correlated with fMRI BOLD signal change in this area during a verbal fluency task (Murata et al., 2010) suggesting a relationship between fNIRS haemodynamic responses and cortical function in this region; for this reason we pre-specified analysis of left-sided responses for the Verbal Fluency task. Finally the design of our study means we are not able to exclude order effects with regard to the effect of ECT but this appears unlikely as high consistency in responses has been demonstrated over repeated tests using verbal fluency tasks (Kakimoto et al., 2009; Kono et al., 2007).

In conclusion, we confirmed a reduction in frontal HbO measured using fNIRS during cognitive activation tasks in depressed patients compared with controls although the result was only significant for the working memory task, with the degree of reduction greater with more severe depressive symptoms. During a course of ECT treatment, there was a further reduction in the haemodynamic response to a category verbal fluency task which did not correlate with changes in cognition or mood. These results are consistent with hypotheses of decreased frontal cortex activity in depression and further suppression during ECT, however the uncertainty about the degree to which the haemodynamic responses measured by fNIRS in this study reflect cerebral cortical function rather than changes in extra-cerebral perfusion places a significant caveat on this interpretation. Future developments in portable fNIRS devices will enable the retrieval of cerebral cortical haemodynamic responses by incorporating short-separation channels to detect the extra-cerebral signal (Gagnon et al., 2014). Nevertheless the reproducibility of fNIRS findings in psychiatric disorders using current technology, and evidence for clinical relevance in predicting response to antidepressant treatment in depression, means further research into understanding the biology of fNIRS frontal responses and their potential utility as a biomarker is warranted.

## Supplemental Material

Supplementary_material_(2) – Supplemental material for Frontal haemodynamic responses in depression and the effect of electroconvulsive therapy Click here for additional data file. Supplemental material, Supplementary_material_(2) for Frontal haemodynamic responses in depression and the effect of electroconvulsive therapy by Darragh Downey, Sabrina Brigadoi, Liam Trevithick, Rebecca Elliott, Clare Elwell, R Hamish McAllister-Williams and Ian M Anderson in Journal of Psychopharmacology
