# Supplementary material for: Frontal haemodynamic responses in depression and the effect of electroconvulsive therapy
Source: J Psychopharmacol. 2019 Jun 25;33(8):1003–14. doi: 10.1177/0269881119858313 (PMC6628632; doi:10.1177/0269881119858313)
Supplement: Supplementary_material_(2) – Supplemental material for Frontal haemodynamic responses in depression and the effect of electroconvulsive therapy [file Supplementary_material_(2).pdf]

Supplementary material

Frontal cortical function in depression and the effect of electroconvulsive therapy

Darragh Downey,[1ꞋI] Sabrina Brigadoi,[2,3Ɨ] Liam Trevithick,[4,5] Rebecca Elliott,[6] Clare Elwell,[2] R Hamish McAllister-Williams,[4,7] Ian M Anderson,[6]*

1 Faculty of Biology, Medicine and Health, The University of Manchester, Manchester, UK
2 Biomedical Optics Research Laboratory, University College London, London, UK
3 Department of Developmental and Social Psychology, University of Padova, Padova, Italy
4 School of Psychology, Newcastle University, Newcastle upon Tyne, UK
5 Northumberland Tyne and Wear NHS Foundation Trust, Newcastle upon Tyne, UK
6 Neuroscience and Psychiatry Unit, Department of Neuroscience and Experimental Psychology, The University of Manchester, UK
7 Institute of Neuroscience, Newcastle University, Newcastle upon Tyne, UK

Ɨ Joint first author

*** Corresponding Author:**
Ian M Anderson, Professor Emeritus of Psychiatry, Neuroscience and Psychiatry Unit, The University of Manchester and Manchester Academic Health Science Centre, Room G809, Stopford Building, Oxford Road, Manchester, M13 9PT, UK
Tel: +44 (0)161 275 7428
Email: ian.anderson@manchester.ac.uk

Requests for accessing original data or analysis methods should be sent to the corresponding author.

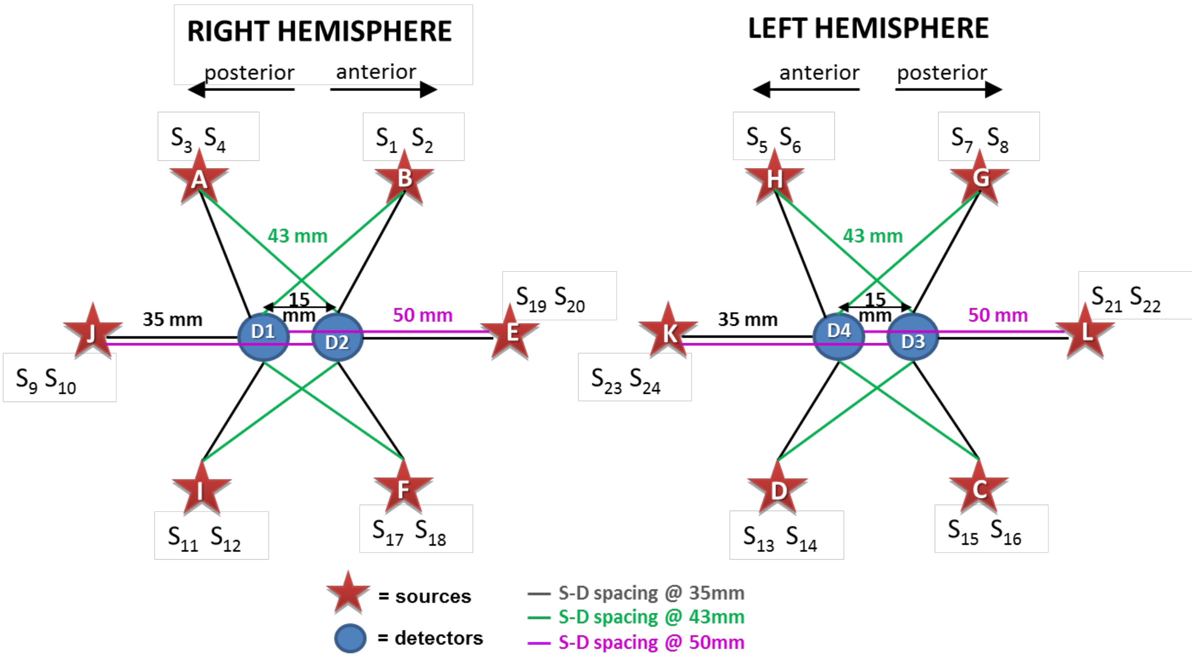

Figure S1: The near-infrared spectroscopy array layout used in the study (viewed from the front).

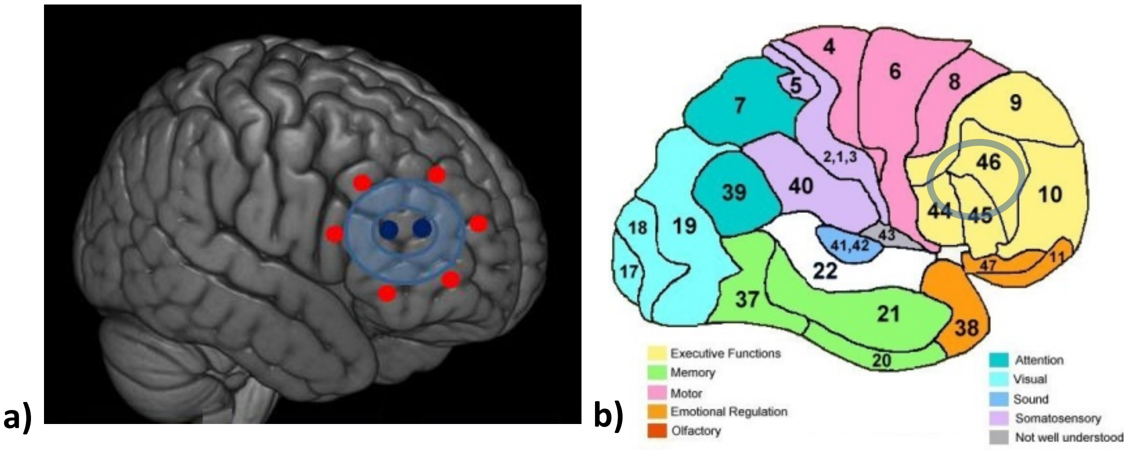

Figure S2: The areas of prefrontal cortex covered by the array.

The left hand panel a) shows a surface render of the fiducial markers on the right side with a shaded 'doughnut ring' from where fNIRS signal was recorded. In panel b) the shaded ring indicates the approximate Brodmann areas corresponding to the area of signal acquisition. (Brodmann areas map reproduced with permission from BrainMaster Technologies Inc (www.brainm.com), Brodmann Atlas)

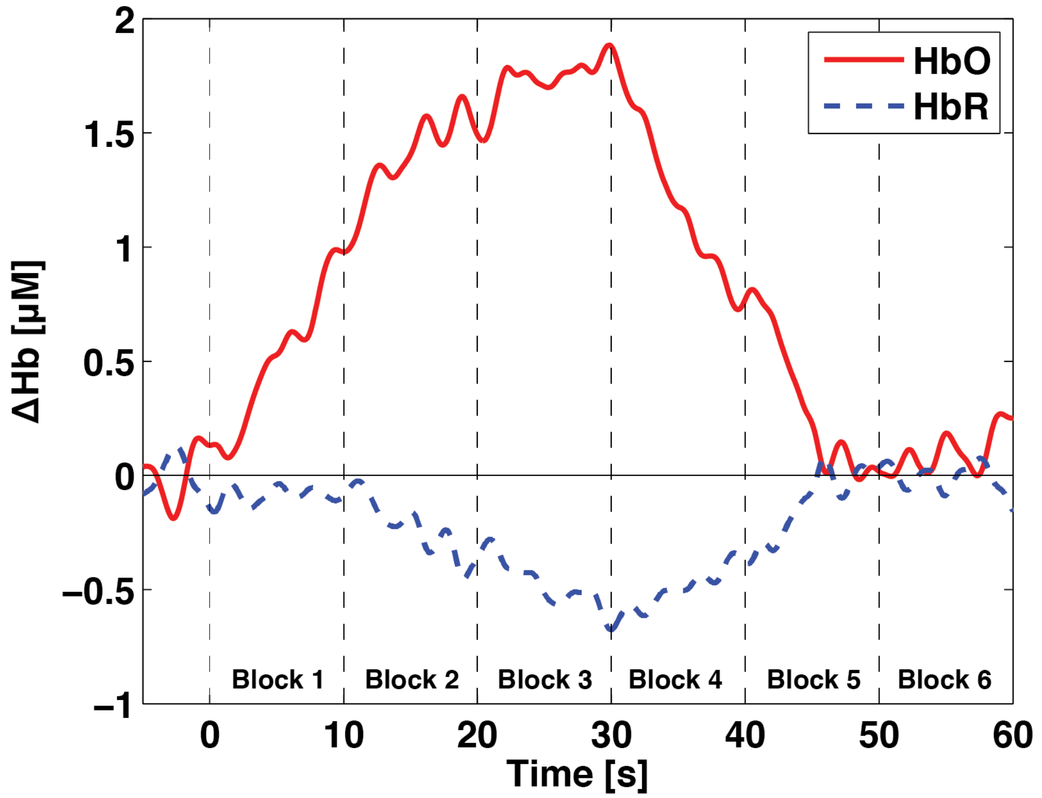

Figure S3: Example of mean haemodynamic response and Block timing.

Shown is the timing of the six 10s blocks used in the analysis (Block 1: 0 - 10s, Block 2: 10 - 20s, Block 3: 20 - 30s etc.). The value of the integral in each block was used for fNIRS analysis.
